# Patient-reported improvements from use of IMC-2 alone and IMC-2 and Paxlovid^®^ in a Long COVID cohort: a case series

**DOI:** 10.3389/fimmu.2025.1698271

**Published:** 2026-01-05

**Authors:** William L. Pridgen, David Putrino

**Affiliations:** 1 Tuscaloosa Surgical Associates, Tuscaloosa, AL, United States; 2 Cohen Center for Recovery from Complex Chronic Illness, Icahn School of Medicine at Mount Sinai, New York, NY, United States

**Keywords:** antiviral, cognitive, dysautonomia, fatigue, infection-associated chronic conditions and illnesses (IACCI), Long COVID, Paxlovid, post-acute sequelae of COVID (PASC)

## Abstract

**Introduction:**

Long COVID (LC) is an infection-associated chronic condition and illness (IACCI) with no currently approved treatments. In order to address SARS-CoV-2 persistence and herpesvirus reactivation, which have been implicated as drivers of LC, sustained use of antiviral combinations may be useful in treating patients with the illness.

**Methods:**

A convenience sample of patients undergoing an extended course of antiviral therapy was studied. Patients received either 120 days of IMC-2 only (IO) or 120 days of IMC-2 with the addition of 15 days of Paxlovid (IP), prescribed off-label at an outpatient clinic for people with LC. The Patient Global Impression of Change (PGIC) was used to measure therapy response over time, with primary focus on fatigue and secondary focus on brain fog and dysautonomia. Visual analog scales (VAS) were also used to track perceived symptom improvements.

**Results:**

A total of 27 people with LC were approached for treatment, of whom 24 completed one or both protocols. Twelve received the IO protocol, and 12 received the continuous IP combination. Both groups reported reductions in fatigue on the PGIC, but participants receiving IP experienced a statistically significant improvement compared with those receiving IO (*p* < 0.0001). Similarly, using a VAS, patients in the IP group reported an average 55.3% (*p* < 0.0001) greater reduction in fatigue than the IO group. Participants who completed the IP intervention demonstrated durable clinical benefit, with symptom improvements remaining consistent at 120-, 305-, and 731-day follow-ups.

**Discussion:**

This small, open-label case series provides pilot evidence supporting the need for a larger trial of combination antivirals for people living with LC. Based on these results, a larger, controlled trial of IMC-2 paired with Paxlovid is recommended.

## Introduction

A subset of individuals infected with the severe acute respiratory syndrome coronavirus 2 (SARS-CoV-2) virus develop new symptoms or sequelae that persist for months or years. This condition is known as long coronavirus disease (COVID) syndrome (LC), post-COVID syndrome, or postacute sequelae of COVID (PASC). Conservative estimates suggest that at least 65 million people worldwide are affected by LC, with some sources indicating that the actual number may be higher (1, 2). The societal and financial consequences of untreated LC have been estimated at nearly three trillion dollars in the USA alone (3). There are likely multiple drivers of LC symptoms, with two of the most prominent being persisting reservoirs of SARS-CoV-2 and SARS-CoV-2-related downregulation of the immune response, which can result in the reactivation of dormant viruses such as the herpesviruses (4, 5). Chronically persistent pathogens likely require different interventional strategies than infections involving acute pathogens, and few existing antiviral clinical trials targeting LC populations have directly addressed this issue (6). In acknowledgment of the literature highlighting the possibility of SARS-CoV-2 persistence as a primary driver of symptomatology, the term “Long COVID (LC)” will be used throughout this manuscript.

To date, only clinical trials addressing potential SARS-CoV-2 persistence in LC with monotherapeutic antivirals exist. Multiple 15- and 30-day Paxlovid monotherapy studies in LC failed to show efficacy but support the drug’s tolerability and safety at these dosages. Similarly, the RECOVER Initiative is currently running 15- and 25-day Paxlovid studies (7, 8). Remdesivir is presently being studied in Finland, and still other researchers are investigating the efficacy of monoclonal antibodies for an LC indication (9). In addition to the possibility of viral persistence, a proportion of LC patients may experience significant symptom burden as a result of persistent or reactivating herpesviruses (10, 11).

To date, all of the reported antiviral trials have tested only single antiviral agents. However, it has been suggested that treating SARS-CoV-2 persistence may require the synergistic use of agents with different mechanisms of action or trials employing longer antiviral dosing periods (6). The question of whether combining antivirals could more effectively target persistent SARS-CoV-2 infection alongside reactivation of other latent viruses is highly relevant to improving care for people with LC. IMC-2, a novel combination of valacyclovir and celecoxib, may have utility in addressing persistent or reactivating herpesviruses and could be relevant to the clinical care of people with LC. Valacyclovir’s antiviral properties are well established, and celecoxib also exhibits strong antiviral properties (12–17). Specifically, both gammaherpesviruses and alphaherpesviruses upregulate Cyclooxygenase (COX)-2, and to a lesser degree, COX-1 enzymes. When administered at concentrations sufficient to inhibit both COX-1 and COX-2 enzymes, celecoxib can serve as an effective antiviral complement to valacyclovir. Trials utilizing celecoxib alone have demonstrated efficacy in treating acute SARS-CoV-2 infections (18). Similarly, Paxlovid, due to its 3C-like (cysteine) proteases, may exhibit activity against more than a dozen viruses in addition to SARS-CoV-2 (19, 20). Therefore, a combination therapy such as IMC-2, with or without Paxlovid, could potentially provide continuous daily suppression of multiple pathogens, reversing viral reactivation of herpesviruses and potentially other viruses.

In the present work, a case series is described in which patients with LC received either IMC-2 only (IO) or IMC-2 combined with a 15-day course of Paxlovid (IP). Outcomes between the two groups are compared and contrasted, and the feasibility of these data to justify a larger placebo-controlled, randomized controlled trial is discussed.

## Methods

All patients seeking care at an LC clinic who were prescribed either the IO and IP medication combinations provided prospective, informed consent for their data to be used for both retrospective review and quality improvement purposes. Ethics approval for the analysis and publication of these data was provided by the Institute of Regenerative and Cellular Medicine (IRCM) Office for Human Research Protection of HHS (IRB Approval Number: IRCM-2025-446). Data were collected in accordance with the Declaration of Helsinki and the FDA’s Good Clinical Practice recommendations. The privacy rights of human subjects were maintained, and appropriate clinical oversight was ensured.

### Participant inclusion

All patient data presented in this case series came from individuals who met the LC diagnostic criteria according to the Centers for Disease Control (CDC) and National Academies of Science, Engineering, and Medicine (NASEM) clinical case definitions. Patients of all genders, over the age of 18, and symptomatic with LC for at least 5 months, were given the opportunity to contribute data. All participants were screened for potential contraindications to the antiviral protocols, including, but not limited to, monitoring for renal and hepatic dysfunction, gastrointestinal bleeding risk, and other comorbidities or medications that could interact with the prescribed medications.

### Symptom rating

Symptom changes were measured using only subjective patient reporting. All patients were asked to rate the severity of three symptoms—fatigue, brain fog, and dysautonomia (dizziness and tachycardia)—using a five-point Likert scale (none, mild, moderate, moderately severe, severe) at the time of enrollment. Subsequently, percentage improvement of these symptoms, as well as a Patient Global Impression of Change (PGIC) at three posttreatment timepoints (120, 305, and 731 days following commencement of the protocol), were also collected. The PGIC was presented as two scales: a seven- and a 10-point scale, where patients were asked to rate their perceived improvement at each posttreatment timepoint. Possible responses on the seven-point PGIC scale ranged from 1 (no change, or condition has gotten worse) to 7 (a great deal better, a considerable improvement that has made all the difference). The 10-point PGIC scale was presented as a visual Analog scale (VAS) asking about symptom severity and ranging from − 5 (much worse) to 5 (much better), with a central point of “no change” at 0. The PGIC has been well validated as a patient-reported outcome that can capture meaningful subjective change in chronic symptom severity (21). Although it has not been specifically validated in LC, it has been well-used and validated in other complex chronic illnesses, such as fibromyalgia, and was therefore selected as a reasonable endpoint to measure self-reported symptom change in the patient sample (22). The primary endpoint of this work was the PGIC in fatigue at 120 days following commencement of the protocol (at the conclusion of the IO and IP protocols). Secondary endpoints included changes in PGIC scale data for brain fog and dysautonomia symptoms at 120 days from commencement of the protocol, as well as changes in PGIC scale data for fatigue, brain fog, and dysautonomia symptoms at 305 and 731 days from commencement of the protocol.

### Intervention

**Figure 1:**
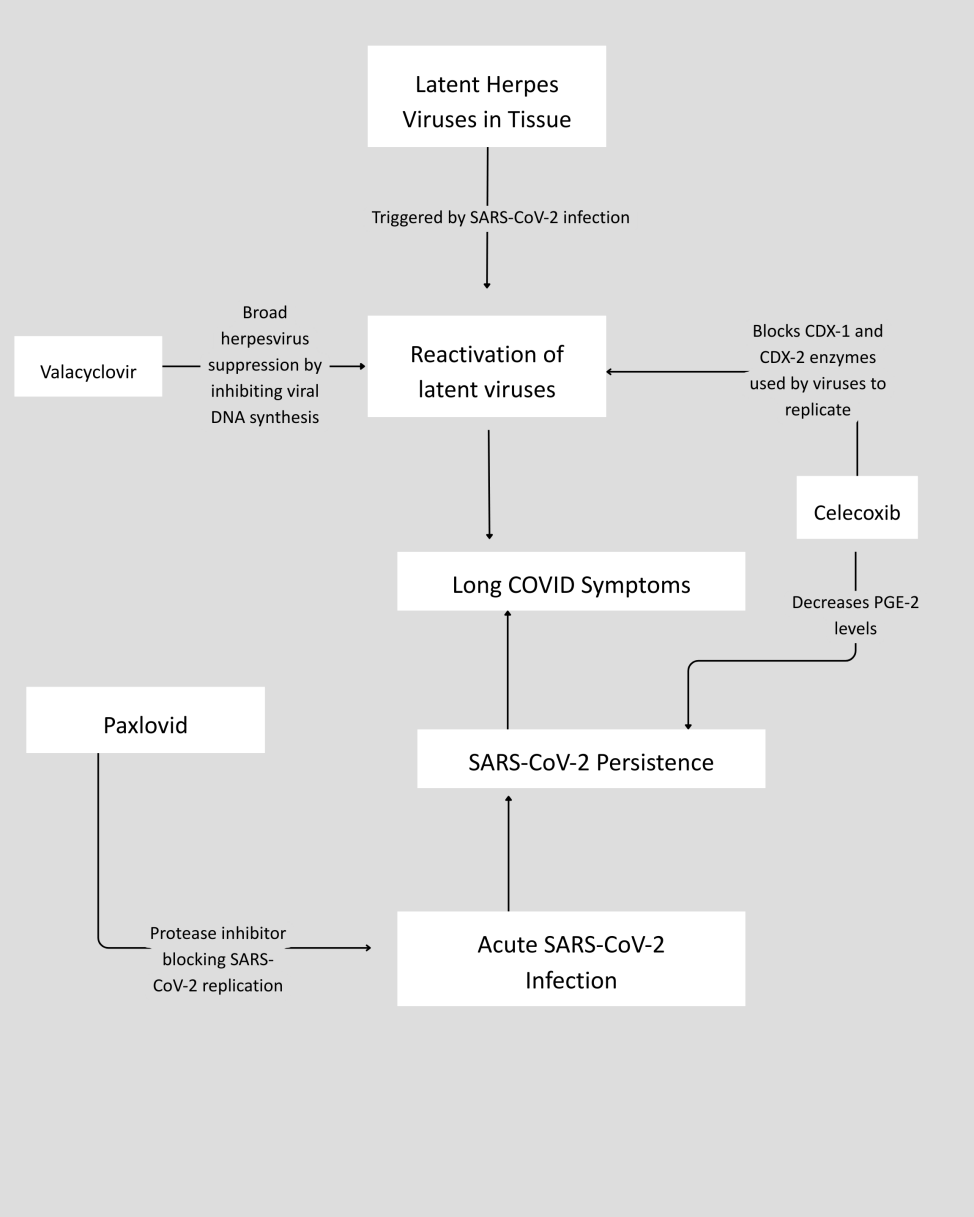
Proposed mechanism of action for the IO and IP component drugs.

This was a single-center, open-label, prospective case series that tracked patient responses to IMC-2 and Paxlovid and included two arms with the opportunity for crossover. Patients who consented to off-label use of IMC-2 to treat Long COVID symptoms could take IMC-2 alone (IO) or combine it with a 15-day Paxlovid course (IP). Patients who initially opted for IO could crossover to IP after completing the 120-day IO protocol. The proposed mechanism of action of this novel drug combination is shown in **Figure 1**. The IO protocol consisted of 1,500 mg of valacyclovir taken twice daily (BID) combined with 200 mg of celecoxib taken BID for 120 days. The IP protocol also consisted of 1,500 mg of valacyclovir taken BID combined with 200 mg of celecoxib taken BID for 120 days. However, during days 13–28 of the protocol, 15 days of Paxlovid were administered. During this 15-day period, the valacyclovir dosage was reduced to 750 mg BID. As part of general clinical safety monitoring, the prescribing physician ordered bloodwork to track kidney and liver function prior to initiating both the IO and IP protocols, as well as every 6–8 weeks while patients were taking their prescriptions. If kidney or liver function became abnormal at any time, medications were discontinued, and follow-up testing continued until kidney and liver function normalized.

### Statistical analysis

Between-group comparisons of symptom improvements, VAS, and PGIC data at different timepoints were conducted using Mann–Whitney *U* (Wilcoxon rank-sum) tests. Effect size was estimated by computing Cohen’s d to describe the magnitude of the difference between the IO and IP groups. Within-group comparisons of symptom improvement and PGIC data at different timepoints were conducted using a Friedman test. Statistical adjustment for multiple comparisons in the secondary outcome analyses was performed using the Bonferroni correction. All analyses were performed in MATLAB (Mathworks, Natick, MA, USA).

## Results

### Patient characteristics

**Figure 2:**
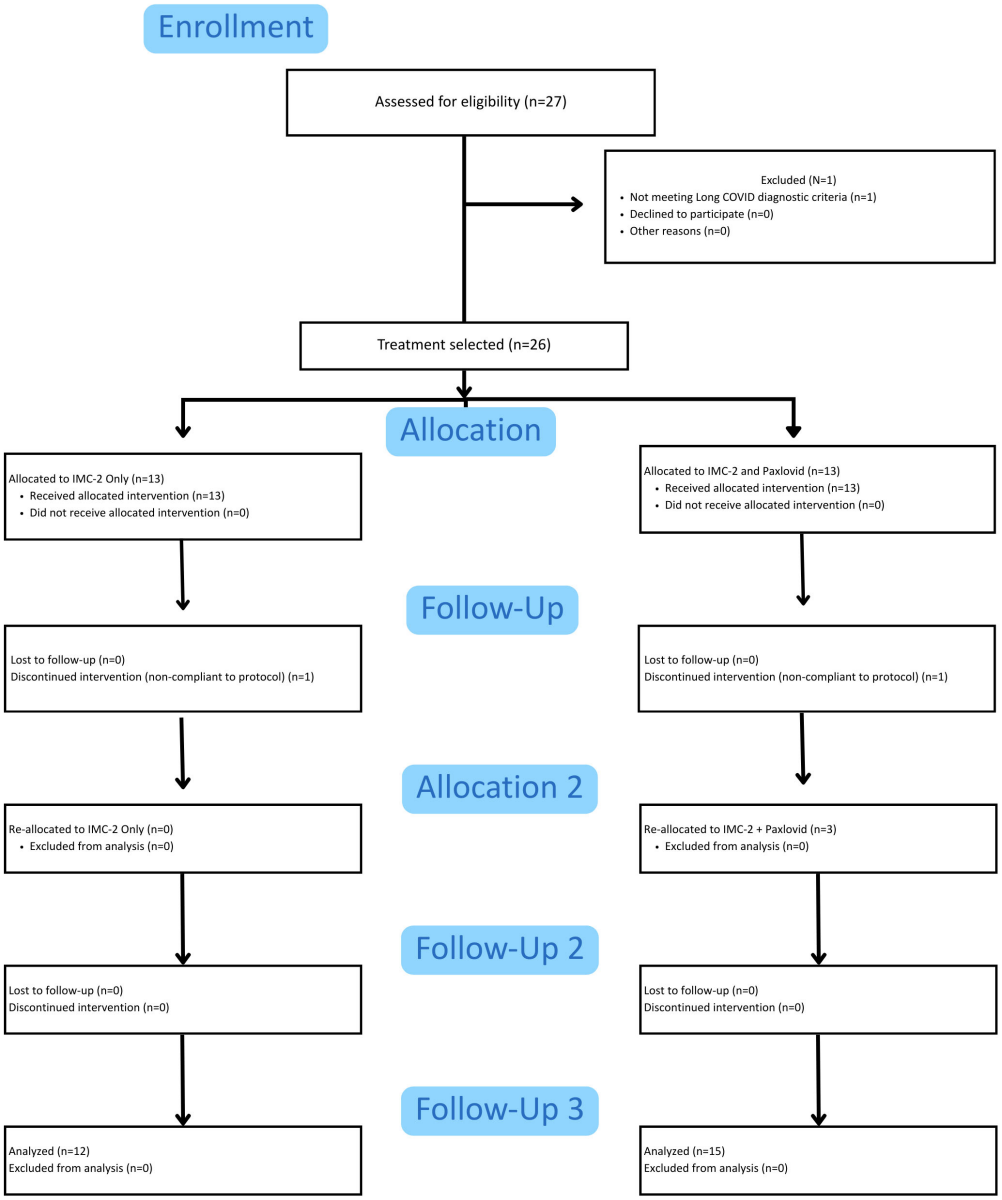
CONSORT diagram of case series patients.

Data presented in this case series were collected from patients with Long COVID attending a single Alabama-based clinic from April 2022 to February 2024. During this period, a total of 27 patients were approached with the opportunity to receive either IO or IP therapy. Twenty-four patients (four men) completed one or both interventional arms. A CONSORT-format flow diagram illustrating patient participation in the different interventional arms is presented in **Figure 2**. Initially, 13 patients opted for IO therapy (two men) and 13 patients (three men) opted for IP therapy, but one patient in each group was noncompliant with the protocol and was subsequently removed from the analysis. In addition, three patients (one man) who initially engaged in IO therapy also opted to attempt the IP combination. In total, 24 patients (12 IO, 12 IP) initially completed the protocols, including three crossovers. Participants in the IO and IP groups were comparable in baseline characteristics, with no differences between the two groups (**Table 1**).

**Table 1:** Baseline Characteristics of IO and IP patients.

Baseline characteristic	IO patients	IP patients
Female (male)	10 (2)	9 (3)
Average age (SD)	52 (15.6)	56 (11.3)
Race/Ethnicity	White (11), Asian (1)/Not Hispanic/Latino (12)	White (11), Black or African American (1)/not Hispanic/Latino (12)
Time from initial infection to initiation of protocol	407 (251.6)	357.2 (256.0)
Number of patients with known reinfections	8	8
Pre-COVID comorbidities	Insomnia (1), chronic constipation (1), IBS (1), fibromyalgia (1)	Insomnia (2), IBS (2), fibromyalgia (1)

### Adverse event and side effect reporting

No side effects or adverse events were reported for patients who received the IO treatment combination. For those who received the IP treatment combination, some minor side effects were reported (**Table 2**), most commonly increased fatigue (40% of patients) and dysgeusia (86.7% of patients). These minor side effects appeared during the initiation of the 15-day Paxlovid course and resolved shortly after its completion.

**Table 2:** Summary of side effect profiles of IO and IP treatment groups.

Side effect	Percentage reported in the IO group	Percentage reported in the IP group
Body aches/Discomfort	0	26.6%
Flu-like symptoms	0	13.3%
Depression	0	13.3%
Headaches/Dizziness	0	13.3%
Diarrhea	0	13.3%
Increased fatigue	0	40%
Bad taste in the mouth	0	86.7%

### Comparison of IO and IP outcomes after 120 days of treatment

**Figure 3:**
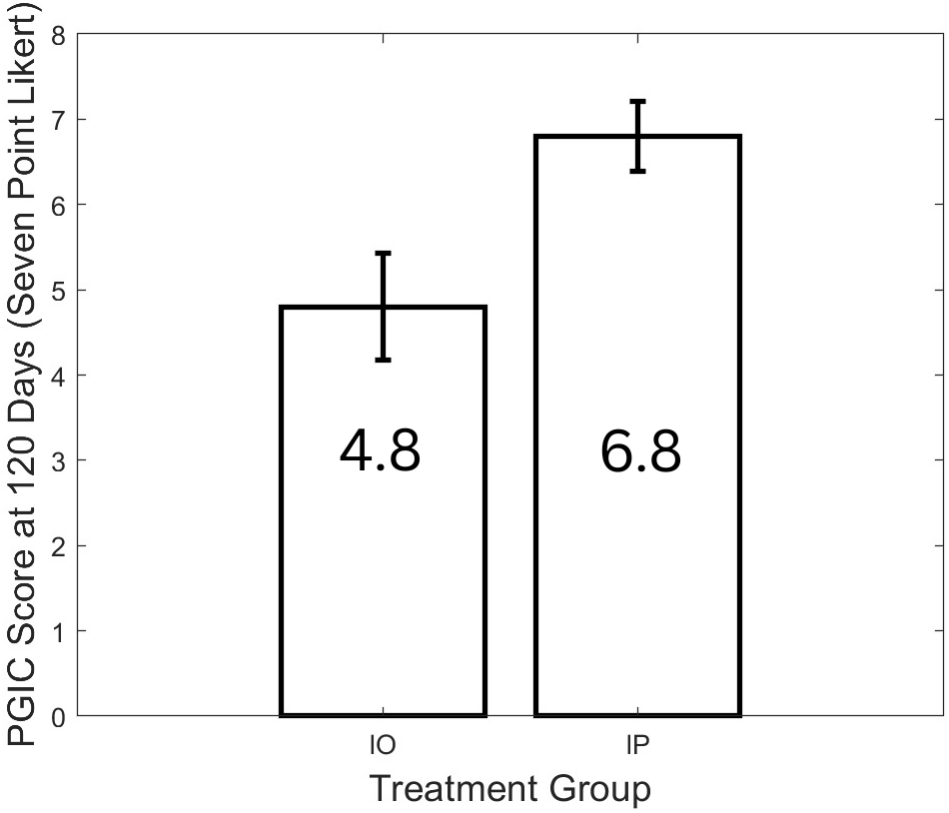
PGIC for fatigue improvement at day 120 for IO compared with IP.

The primary endpoint was to determine whether a significant change in fatigue occurred when 15 days of Paxlovid were given in combination with 120 days of valacyclovir and celecoxib, compared to 120 days of valacyclovir and celecoxib alone. On average, patients who completed the IP protocol scored 2 points higher on their PGIC fatigue rating than those on the IO protocol (**Figure 3**; *p* < 0.0001, Cohen’s d = 1.8). Additional outcomes between the two groups showed a similar trend of statistically significant improvement on the IP protocol and are listed in **Table 3**.

**Table 3:** Differences in day 120 outcomes between the two protocols.

Outcome	IO average reported score (± standard deviation)	IP average reported score (± standard deviation)	Mean difference (Cohen’s d)	*p*-value
Fatigue (PGIC: 7-point Likert)	4.8 (± 0.63)	6.8 (± 0.4)	IP response is 2 points greater (1.8)	< 0.0001
Dysautonomia (PGIC: 7-point Likert)	4.1 (± 2.3)	6.9 (± 0.3)	IP response is 2.8 points greater (1.4)	< 0.001
Brain fog (PGIC: 7-point Likert)	4.2 (± 1.7)	6.4 (± 0.7)	IP response is 2.2 points greater (1.4)	< 0.001
Fatigue (PGIC: 10-point Likert)	2.3 (± 0.9)	4.0 (± 1.3)	IP response is 1.7 points greater (1.2)	< 0.002
Dysautonomia (PGIC: 10-point Likert)	1.8 (± 3.2)	4.1 (± 1.5)	IP response is 2.6 points greater (1.2)	< 0.05
Brain fog (PGIC: 10-point Likert)	2.5 (± 1.3)	3.8 (± 1.5)	IP response is 1.2 points greater (0.8)	0.06

### Durability of treatment response in the IP cohort

**Figure 4:**
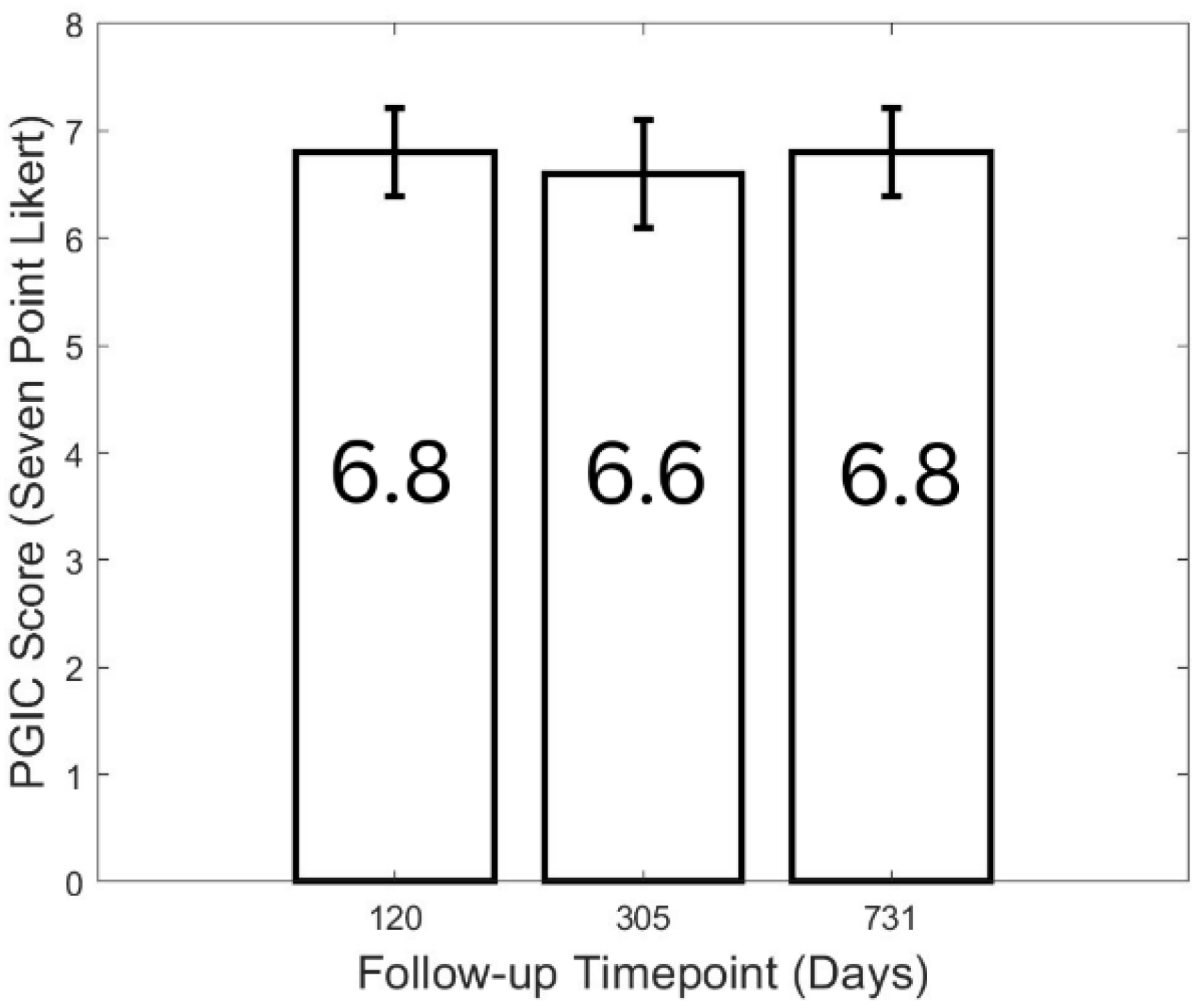
PGIC for fatigue improvement for patients on the IP protocol at 120-, 305-, and 731-day follow-up timepoints.

The durability of each patient’s treatment response to the IP intervention was tracked for 600 days beyond the initial 120-day protocol. All 15 participants in the IP group completed all three follow-up timepoints. No statistically significant differences were observed in symptom reporting for the primary outcome measure (**Figure 4**) or any of the assessed symptoms (**Table 4**) at any of the three timepoints, indicating that treatment responses produced sustained improvements lasting up to 600 days after the cessation of the combined antiviral protocol.

**Table 4:** Durability of treatment responses for patients on the IP protocol up to 731 days.

Outcome	120-day follow-up	305-day follow-up	731-day follow-up	*p*-value
Fatigue (PGIC: 7-point Likert)	6.8 (± 0.4)	6.6 (± 0.5)	6.8 (± 0.4)	> 0.05
Dysautonomia (PGIC: 7-point Likert)	6.9 (± 0.3)	6.6 (± 0.7)	6.8 (± 0.4)	> 0.05
Brain fog (PGIC: 7-point Likert)	6.4 (± 0.7)	6.8 (± 0.4)	6.8 (± 0.6)	> 0.05
Fatigue (PGIC: 10-point Likert)	4.0 (± 1.3)	4.0 (± 1.1)	3.8 (± 1.3)	> 0.05
Dysautonomia (PGIC: 10-point Likert)	4.1 (± 1.5)	4.1 (± 1.2)	4.5 (± 1.1)	> 0.05
Brain fog (PGIC: 10-point Likert)	3.8 (± 1.5)	4.1 (± 1.4)	4.1 (± 1.4)	> 0.05

### Responses of crossover patients

Three patients engaged in both IO and IP interventions. All three patients first completed the IO protocol and then completed the IP protocol following a washout period (range: 23–188 days). **Table 5** presents self-reported outcomes for each crossover patient, including fatigue, dysautonomia, and cognitive symptoms.

**Table 5:** Individual treatment responses for crossover patients.

Patient number	IO PGIC day 120 (washout period)	IP PGIC day 120	IP PGIC day 305	IP PGIC day 731
Fatigue				
1	4 (188)	7	7	7
2	5 (23)	7	7	7
3	5 (118)	7	6	7
Dysautonomia				
1	1	7	7	7
2	5	7	7	7
3	2	6	6	6
Cognition				
1	4	5	7	7
2	2	7	7	7
3	1	6	7	7

## Discussion

This initial open-label case series shows that Long COVID patients treated with either IMC-2 alone or in combination with Paxlovid reported significant improvements in their symptoms, with those receiving the combination therapy experiencing the greatest benefit over a prolonged follow-up. The reported benefits suggest durability of the protocol, as the majority of patients continued to experience improvements in fatigue, dysautonomia, and cognitive symptoms over 600 days postprotocol. In addition, the combination therapy was well tolerated, with minimal adverse events reported and no dropouts related to side effects. These findings provide promising preliminary evidence of feasibility and efficacy, highlighting the potential utility of combination antiviral therapies for a subset of Long COVID and provide rationale and feasibility for a larger, randomized, double-blind, placebo-controlled trial.

There are various reasons why a combination of celecoxib, valacyclovir, and Paxlovid is well-positioned as a therapy to address symptoms arising from Long COVID. Evidence from acute COVID-19 indicates that the SARS-CoV-2 virus can trigger a prostaglandin E2 (PGE2) storm in a substantial proportion of patients via upregulating cyclooxygenase-2 (COX-2) and downregulating PGE2-degrading enzymes within host cells. Treatment with 200 mg twice a day was associated with a lower mortality rate in COVID-19 compared to a control group (18). Upregulated COX-2 levels can also influence events in the Epstein–Barr virus life cycle related to latency–lytic reactivation (12). In addition to gammaherpesviruses, alphaherpesviruses also upregulate COX-2 and, to a lesser extent, COX-1 enzymes. Therefore, the ability of celecoxib, at the proper concentration, to block both COX-1 and COX-2 enzymes makes it an effective antiviral complement to valacyclovir in combating herpesvirus reactivation.

Paxlovid, a combination of nirmatrelvir and ritonavir, is active against SARS-CoV-2 and potentially against other reactivating viruses. Nirmatrelvir, a protease inhibitor, inhibits viral replication by cleaving viral polyproteins involved in replication. Ritonavir has no activity against SARS-CoV-2, but it specifically inhibits the metabolism of nirmatrelvir by cytochrome P450-3A (CYP3A), thereby boosting its potency. However, Paxlovid, due to its 3C-like proteases (cysteine) activity, potentially acts against more than a dozen viruses beyond SARS-CoV-2 (19, 20).

Both latent pathogen reactivation and viral persistence have been implicated multiple times as potential drivers of symptoms in Long COVID (4–6, 23). In addition, prolonged pathogen persistence and reactivation may cause downstream immunopathology. Participants in this case series had been symptomatic for over a year, and persistent immune activation for this duration could cause immune sequelae such as T-cell exhaustion, altered interferon signaling, and altered B-cell function, which have been previously described as potential contributors to symptom burden in people with Long COVID (4, 6). These drivers can occur in combination or in isolation in different individuals, but a large proportion of people with Long COVID are likely experiencing severe symptoms as a result of these mechanisms, which could potentially be treated by directly addressing pathogen persistence and reactivation. Taken together, this provides a strong rationale for why many patients in this case series responded positively to this combination of antiviral medications.

### Limitations

There are several limitations to this study. It was an open-label case series, in which both selection bias—arising from patients actively seeking care—and the placebo effect may have influenced the observed outcomes. In addition, the data show a pronounced bias toward female patients. Although this is consistent with reported gender differences in Long COVID and the likelihood of clinical presentation in outpatient settings, it limits the generalizability of these findings. Finally, aside from using the PGIC and VAS methodologies to assess symptom improvement, symptom severity was not measured with gold-standard instruments but relied on subjective patient-reported ratings.

## Conclusion

The results of this case series suggest a potential synergy between IMC-2 and Paxlovid, as indicated by an apparent improvement in antiviral performance with the combination compared to IMC-2 alone in this pilot study. There are substantial limitations inherent to a single-site, open-label, retrospective review of data of this type of data; however, these early findings are sufficiently promising to justify a larger clinical trial. A randomized, double-blind, placebo-controlled trial is the necessary next step to validate this therapeutic antiviral combination.

## Data Availability

The raw data supporting the conclusions of this article will be made available by the authors, without undue reservation.
